# Effects of surgical specialization and surgeon resection volume on postoperative complications and mortality rate after emergent colon cancer resection

**DOI:** 10.1093/bjsopen/zrad033

**Published:** 2023-05-09

**Authors:** Jenny Engdahl, Astrid Öberg, Henrik Bergenfeldt, Marcus Edelhamre, Tomas Vedin, Sandra Bech-Larsen, Stefan Öberg

**Affiliations:** Department of Surgery, Helsingborg Hospital, Clinical Sciences Lund, Lund University, Lund, Sweden; Department of Surgery, Helsingborg Hospital, Clinical Sciences Lund, Lund University, Lund, Sweden; Department of Clinical Sciences, Lund University, Lund, Sweden; Department of Surgery, Helsingborg Hospital, Clinical Sciences Lund, Lund University, Lund, Sweden; Department of Clinical Sciences, Lund University, Lund, Sweden; Department of Surgery, Helsingborg Hospital, Clinical Sciences Lund, Lund University, Lund, Sweden; Department of Surgery, Helsingborg Hospital, Clinical Sciences Lund, Lund University, Lund, Sweden

## Abstract

**Background:**

The aim of this study was to evaluate the effect of surgical specialization and surgeon resection volume on short-term outcome after emergent colon cancer resections.

**Methods:**

A retrospective analysis of all patients who underwent resections for colon cancer between 2011 and 2020 at Helsingborg Hospital, Sweden was performed. The senior surgeon participating in each procedure was classified as a colorectal surgeon or a non-colorectal surgeon. Non-colorectal surgeons were further divided into acute care surgeons or surgeons with other specialties. Surgeons were also divided into three groups based on median yearly resection volumes. Postoperative complications and 30- or 90-day mortality rate after emergent colon cancer resections were compared in patients operated on by surgeons with different specializations and yearly resection volumes.

**Results:**

Of 1121 patients resected for colon cancer, 235 (21.0 per cent) had emergent procedures. The complication rate of emergent resections was similar in patients operated on by colorectal surgeons and non-colorectal surgeons (54.1 *versus* 51.1 per cent respectively), and the subgroup of acute care surgeons (45.8 per cent), whereas resections performed by general surgeons were significantly associated with more frequent complications (odds ratio (OR) 2.5 (95 per cent c.i. 1.1 to 6.1)). The complication rate was numerically highest in patients operated on by surgeons with the highest resection volumes, which differed significantly from that of surgeons with intermediate resection volumes (OR 4.2 (95 per cent c.i. 1.1 to 16.0)). There was no difference in the mortality rate of patients operated on by surgeons with different specializations or yearly resection volumes.

**Conclusion:**

This study documented similar morbidity and mortality rates after emergent colon resection performed by colorectal and acute care surgeons, but patients operated on by general surgeons had more frequent complications.

## Teaser

The effect of surgical specialization and surgeon resection volume on short-term outcome after emergent colon cancer resection was retrospectively studied. Colon resections were performed with similar quality and comparable rates of morbidity and mortality by colorectal surgeons and non-colorectal surgeons.

## Introduction

Colorectal cancer remains one of the most prevalent malignancies worldwide and a leading cause of cancer-related death^1^. The fact that up to 30 per cent of colorectal cancer patients present with acute symptoms due to mechanical obstruction or perforation is challenging as emergent colon resections are associated with significantly higher morbidity and mortality rates compared with elective surgery^2–4^. The relatively poor results after emergent colon resections may be explained by the fact that these patients generally are older, have a more advanced stage of disease, more frequently suffer from medical co-morbidities, and are in a poorer physiological condition^5^.

There are many reports indicating that patients undergoing elective colon resections performed by surgeons who are specialized in colorectal surgery have lower morbidity rate and postoperative mortality rate than patients operated on by non-colorectal surgeons (NCS)^6,7^. Although there is no proof of a causal relationship between surgical specialization and better surgical outcome, it seems logical that frequent training and high volumes of elective surgery may result in fewer and less severe complications, and lower mortality rates. However, the advantageous results from elective procedures cannot automatically be extrapolated for emergent colon resections. The management of patients in need of emergent colon resections for cancer is more challenging than that of patients who undergo elective resections. It is therefore important that emergent colon cancer surgery is performed by surgeons who are appropriately trained. The first challenge is to manage the acute surgical problem in patients in a poor physiological condition due to perforations or mechanical obstructions to minimize the morbidity and mortality rates after surgery. The second challenge is to perform surgery with high oncological quality, to reduce the risk of local recurrence of disease and to achieve the best long-term survival for the patient. Theoretically, specialized colorectal surgeons (CS) may be better trained and have more experience in performing adequate oncological surgery with proper resection margins and adequate lymph node dissection. However, acute care surgeons (ACS) may have more experience in managing acute surgical problems in critically ill patients. As patients requiring emergent surgery for colon cancer need surgeons with a combination of these skills, the question of whether the qualification of surgeons with a certain specialization in general is better suited for this type of surgery arises. Although there are some reports suggesting that high-volume specialized CS have lower morbidity and mortality rates after emergent resections for colon cancer^8^, there are numerous studies showing similar outcomes for patients having emergent colon resection performed by CS and NCS^9–14^. Thus, the importance and the effect of surgical specialization and surgeon resection volume on complications and mortality rate after emergent resections for colon cancer remain unclear.

The aim of this retrospective study was to evaluate and compare the effect of surgical specialization and surgeon resection volume on early postoperative outcome after emergent colon cancer resections at a secondary care hospital.

## Methods

### Study population

The study population consisted of patients who underwent emergent and elective resections for colon cancer between 2010 and 2020 at Helsingborg Hospital, Sweden, which is a secondary care hospital with a catchment area consisting of 350 000 residents. The patients were identified from the Swedish Colorectal Cancer Registry (SCRCR), which is a prospective and validated national registry with a coverage of 98.5 per cent for colon cancer^15^. Patients with a tumour located within 15 cm of the anal verge and cancer originating from the appendix were not included in the study. Demographic and clinical characteristics (patient sex, age, BMI, and ASA scores), tumour characteristics (TNM stage), surgical data (type of resection, resection margin status, operating time, intraoperative bleeding, and use of stoma), and data from the patients’ post-operative course (intensive care unit (ICU) admission, length of hospital stay, and mortality rate) were extracted from the SCRCR. A retrospective manual review of the patients’ medical records was conducted by three researchers to obtain additional clinical information (co-morbidity scores, indication for emergent surgery, if emergent resections were performed during or outside regular working hours, and the severity of complications according to the Clavien–Dindo classification), and to validate data from the SCRCR. To reduce the risk of information bias during data collection, the guidelines for retrospective medical record reviews were followed^16^.

### Definitions

The Charlson co-morbidity index (CCI) was used to classify the co-morbidity burden of the patients^17^. The stage of colon cancer was categorized from I to IV according to the TNM classification^18^. Surgery was defined as emergent or elective. Emergent surgery was defined by procedures performed within 48 h after acute admission. As the timing of emergent surgery may affect postoperative outcome, a distinction was made between patients having emergent colon resections performed during ordinary working hours and those operated on during on-call hours. The latter were defined as having surgery outside regular working hours. Postoperative complications were characterized and categorized according to the Clavien–Dindo classification^19^. For the purpose of this study only Clavien–Dindo grades II to V were used. Complications were double-checked in medical records and in the SCRCR.

### Surgeons and surgical specialization

The most senior surgeon actively participating in each of the colon resections was noted and classified according to the surgical specialization. When resections were performed by more than one specialist in general surgery, which predominantly occurred during elective surgery, only the most senior surgeon of each procedure was utilized in the estimate. Colon resections performed for non-malignant disease and rectal cancer were not included in the calculation of the yearly resection volumes. Colorectal surgery is not recognized as an official surgical specialization in Sweden and CS were therefore defined as surgeons primarily working at the hospital’s colorectal unit. For the purpose of this study, the surgeons were divided into groups based on their surgical specialization. First, all surgeons were divided into two groups consisting of CS and NCS. Secondly, the group of NCS was further divided into two groups, the first of which consisted of ACS who primarily work with trauma and non-trauma emergency surgery, and a second group consisting of board-certified general surgeons (GS) with additional specialization in upper gastrointestinal surgery or vascular surgery. The latter group of surgeons was for the purpose of this study defined as GS. Two separate analyses were made to assess the effect of surgical specialization on the outcome after emergent colon cancer resections. In the first analysis, the postoperative results were compared between groups of patients operated on by CS and NCS. In an additional analysis the postoperative outcomes were compared between groups of patients operated on by CS, ACS, and GS.

To assess the effect of surgeon colon cancer resection volumes on postoperative outcome, the surgeons were categorized into three groups based on the median and the 75th percentile of their annual resection volumes (elective and emergent) as the most senior surgeon.

### Outcomes of interest

The primary outcome of interest was the occurrence and the severity of postoperative complications according to the Clavien–Dindo classification^19^. The length of hospital stay, the need for reoperations and ICU care, the 30- and 90-day mortality rates, and the proportion of readmissions within 30 days of hospital discharge, as well as the types of surgical complications, were assessed and compared between patients operated on by surgeons with different specializations and different yearly resection volumes, as secondary aims.

### Statistics

Analysis of normality was carried out using the Kolmogorov–Smirnov test. As continuous data were not normally distributed, results are reported as medians and interquartile ranges (i.q.r.), and comparisons were made using non-parametric statistics. The Mann–Whitney *U* test was used to compare continuous data between individual groups and the Kruskal–Wallis test was used to compare multiple groups. Proportions were analysed using the chi-squared test. Logistic regression analysis was used to assess the influence of multiple factors on binary outcomes and is presented using odds ratio (OR) and 95 per cent c.i. Factors that were considered clinically important and believed to potentially effect outcome were entered simultaneously for adjustments in the regression model. Confidence intervals not including 1 and *P* values <0.050 were considered to represent statistical significance. All statistical analyses were performed using SPSS^®^ (IBM, Armonk, NY, USA; Version 25). The study was approved by the Swedish Ethical Review Authority (2019-04329).

## Results

### Study population

In total, 1121 patients underwent colon resections due to colon cancer at Helsingborg Hospital between 2010 and 2020. No patients with a need for emergent colon resection were referred to other hospitals and the postoperative care was provided at the hospital’s unit for colorectal surgery. During the study interval, no patients with obstructing colon cancers were treated with colon stents as a bridge to elective surgery. After data validation, there were no missing data.

Of the colon resections, 886 (79.0 per cent) were elective and 235 (21.0 per cent) were emergent procedures. The emergent resections were performed by 30 different senior surgeons, of which 14 were classified as CS and 16 as NCS. The group of NCS consisted of five ACS and 11 GS. The median annual number of elective and emergent colon resections for cancer performed as the most senior surgeon was 4.1 (i.q.r. 1.2–8.4) per year. For comparison, when all cancer resections performed by the surgeons were included in the annual resection volumes, the total median number of resections was 8.9 (i.q.r. 2.3–18.5) per year. The median total annual resection volume for CS and NCS was 18.7 (i.q.r. 9.8–27.5) and 2.4 (i.q.r. 0.9–6.0) respectively. Ninety-nine (42 per cent) of the patients who underwent emergent surgery had colon resections performed by CS and in 136 (58 per cent) patients the resections were performed by NCS. Based on the median and 75th percentile of the total annual cancer resection volume as the most senior surgeon, 14 of the 30 surgeons had a median yearly resection volume of less than 4.11, and nine of the surgeons performed 4.11–8.40 colon resections for cancer per year. Seven surgeons, all of whom were specialized CS, had a median yearly rate of colon cancer resections exceeding 8.40 per year.

### Outcomes associated with surgical specialization

Clinical characteristics and descriptions of the surgical procedures of patients who underwent emergent resections performed by CS and NCS are shown in *Table S1*. The median age and BMI, the sex distribution, and the co-morbidity burden were similar in the two groups of patients. There was no difference in the indications for emergent colon resections and the type of surgical procedure performed was similar in patients operated on by CS and NCS. Furthermore, there was no difference in the median operating time, the perioperative blood loss, the number of resected lymph nodes, or the tumour stage between the two groups of patients. The proportion of R_1_ resections was numerically lower for patients operated on by NCS, but the difference did not quite reach statistical significance. This was further examined in an adjusted regression analysis, but no significant association between R_1_ resections and colon resections performed by CS was found. In patients with left-sided cancers, the proportion of protective stomas was significantly higher in patients operated on by CS. The frequency and the severity of complications according to the Clavien–Dindo classification was similar in patients operated on by CS and NCS (*Table S2*). The rate of reoperations due to anastomotic leakage in patients operated on by CS and NCS was comparable (3.0 *versus* 2.9 per cent respectively, *P* = 0.968). Similarly, there was no difference in the proportions of patients with intra-abdominal abscesses (3.0 *versus* 1.5 per cent respectively, *P* = 0.423), patients with wound site infections (9.1 *versus* 7.4 per cent respectively, *P* = 0.641), or patients who were reoperated on due to wound dehiscence (4.0 *versus* 2.9 per cent respectively, *P* = 0.646). Furthermore, the length of hospital stay, the frequency of reoperations, the need for ICU postoperative care, and the rates of 30- and 90-day mortality rate were similar in the two groups of patients. However, unplanned hospital readmissions were significantly more frequent in patients operated on by NCS, which was demonstrated by an adjusted OR that was 4.2 times higher than that of patients operated on by CS.

In the data collection and validation process, it was observed that 176 of the 1121 (15.7 per cent) patients who underwent colon resections were misclassified regarding the presence or absence of complications. Of these patients, no complications were registered in the SCRCR, but were present according to the medical records in 152 (86.4 per cent) patients. Conversely, complications were registered in the SCRCR in 24 patients (13.6 per cent), but uneventful postoperative courses without complications were documented in the medical records. In the analysis of postoperative outcome in patients operated on by CS, ACS, and GS, there was no significant difference in the patient characteristics, the indications for emergent surgery, or the types of surgical procedures (*Table 1*). There was a significant difference in the estimated perioperative blood loss, with the highest numerical volume for GS, whereas the operating time, the tumour stage, and the indicators of adequate oncological surgery were similar in patients operated on by the three groups of surgeons. The proportion of complications was numerically lowest in patients operated on by ACS, but the difference did not quite reach statistical significance (*Table 2*). In a complementary binary regression analysis, there was a significant association between complications and emergent colon resection performed by GS, with an adjusted OR that was 2.5 times higher than that of ACS (*Table 2*). No such association was found for patients operated on by CS. The distribution of complications according to the Clavien–Dindo classification was similar in the three groups of patients. The proportion of patients who needed ICU care was numerically lowest for patients operated on by ACS, but there was no significant difference between the groups. The duration of hospital stay was similar in groups of patients operated on by CS, ACS, and GS, but the rate of reoperations differed significantly. Compared with patients operated on by ACS, who had the numerically lowest proportion of reoperations, a binary regression analysis showed a significant association between reoperations and colon resections performed by GS, but no such association was found for resections performed by CS. Similarly, the proportion of unplanned hospital readmissions differed significantly between the three groups of patients. A complementary adjusted binary regression analysis showed a significant association between readmissions and emergent colon resections performed by ACS and GS, with ORs that were 3.5 and 7 times higher respectively than that of patients operated on by CS. There was no difference in the 30- or 90-day mortality rate of patients operated on by the three groups of surgeons.

**Table 1:** Patient characteristics and descriptions of surgical procedures in patients who underwent emergent colon cancer resections performed by colorectal surgeons, acute care surgeons, and general surgeons

	Resections performed by			*P*
	Colorectal surgeons, *n* = 99	Acute care surgeons, *n* = 107	General surgeons, *n* = 29	
**Age (years), median (i.q.r.)**	76 (68–83)	77 (66–84)	81 (72–86)	0.165
**Sex**				0.892
Male	52 (52.5)	54 (50.4)	16 (55.2)	
Female	47 (47.5)	53 (49.5)	13 (44.8)	
BMI (kg/m^2^), median (i.q.r.)	24.3 (21.9–27.5)	23.8 (21.8–27.0)	23.8 (21.3–28.0)	0.819
**ASA classification**				
I	10 (10.1)	16 (15.0)	2 (6.9)	0.348
II	43 (43.4)	48 (44.9)	10 (34.5)	
III	41 (41.4)	38 (35.5)	17 (58.6)	
IV	5 (5.1)	5 (4.7)	0 (0.0)	
**Charlson co-morbidity index**				
2	48 (48.5)	51 (47.7)	9 (31.0)	0.363
3	19 (19.2)	20 (18.7)	10 (34.5)	
4	14 (14.1)	9 (8.4)	5 (17.2)	
5	1 (1.0)	2 (1.9)	1 (3.4)	
6	8 (8.1)	19 (17.8)	2 (6.9)	
7	5 (5.1)	4 (3.7)	2 (6.9)	
≥8	4 (4.0)	2 (1.9)	0 (0.0)	
**Indications for surgery**				
Obstruction	81 (81.8)	89 (83.2)	24 (82.8)	0.592
Perforation	12 (12.1)	11 (10.3)	5 (17.2)	
Anaemia/bleeding	6 (6.1)	7 (6.5)	0 (0)	
**Surgical procedures**				
Right hemicolectomy	50 (50.5)	48 (44.9)	13 (44.8)	0.392
Sigmoid resection	15 (15.2)	21 (19.6)	2 (6.9)	
Colectomy	14 (14.1)	9 (8.4)	6 (20.7)	
Left hemicolectomy	10 (10.1)	18 (16.8)	2 (6.9)	
Hartmann’s resection	7 (7.1)	7 (6.5)	5 (17.2)	
Transverse colon resection	2 (2.0)	3 (2.8)	1 (3.4)	
Low anterior resection	1 (1.0)	1 (0.9)	0 (0)	
Resections outside regular working hours	39 (39.4)	38 (35.5)	15 (51.7)	0.284
Operating time (min), median (i.q.r.)	180 (150–241)	187 (151–230)	196 (150–240)	0.963
Perioperative bleeding (ml), median (i.q.r.)	200 (100–300)	100 (50–200)	300 (150–325)	0.008
Lymph node yield, median (i.q.r.)	27 (21–38)	26 (20–36)	28 (21–40)	0.663
Lymph node yield >12	95 (96.0)	103 (96.3)	28 (96.6)	0.987
R_1_ resections	6 (6.1)	1 (0.9)	1 (3.4)	0.128
**Stoma formation** *				
Permanent stoma	21/52 (40.4)	30/60 (50.0)	10/16 (62.5)	0.266
Protective stoma	6/52 (11.5)	2/60 (3.3)	0/16 (0.0)	0.110
Any stoma	27/52 (51.9)	32/60 (53.3)	10/16 (62.5)	0.754
**Tumour stage**				
I	2 (2.0)	0 (0)	0 (0)	0.601
II	38 (38.4)	42 (39.3)	8 (27.6)	
III	45 (45.5)	49 (45.8)	17 (58.6)	
IV	14 (14.1)	16 (15.0)	4 (13.8)	

Values are *n* (%) unless otherwise indicated. i.q.r., interquartile range.

* Only patients with left-sided tumours included in analysis.

**Table 2:** Postoperative complications and outcome after emergent colon cancer resections performed by colorectal surgeons, acute care surgeons, and general surgeons

	Resections performed by			*P*
	Colorectal surgeons *n* = 99	Acute care surgeons *n* = 107	General surgeons *n* = 29	
**Clavien–Dindo classification of complications**				
No complication	45 (45.5)	58 (54.2)	9 (31.0)	0.349
II	33 (33.3)	29 (27.1)	11 (37.9)	
III	10 (10.1)	7 (6.5)	5 (17.2)	
IV	7 (7.1)	6 (5.6)	1 (3.4)	
V	4 (4.0)	7 (6.5)	3 (10.3)	
**Any complication**	54 (54.5)	49 (45.8)	20 (69.0)	0.073
Regression analysis*, OR (95% c.i.)	1.4 (0.8,2.4)	Reference	2.5 (1.1,6.1)	–
ICU care	18 (18.2)	10 (9.3)	4 (13.8)	0.181
**Reoperations**	12 (12.1)	6 (5.6)	6 (20.7)	0.042
Regression analysis*, OR (95% c.i.)	2.3 (0.7,6.8)	Reference	4.2 (1.1,16.0)	–
Length of hospital stay (days), median (i.q.r.)	13 (9–22)	12 (8–16)	15 (8–32)	0.105
**Readmissions†**	4/95 (4.2)	12/100 (12.0)	6/26 (23.1)	0.011
Regression analysis*, OR (95% c.i.)	Reference	3.5 (1.1,11.6)	7.0 (1.7,29.2)	–
30-day mortality rate	3 (3.0)	7 (6.5)	3 (10.3)	0.262
90-day mortality rate	8 (8.1)	7 (6.5)	3 (10.3)	0.775

Values are *n* (%) unless otherwise indicated. OR, odds ratio; ICU, intensive care unit; i.q.r., interquartile range.

* Binary regression analysis with adjustments for ASA classification, tumour stage, indication for emergent surgery, and surgery performed outside regular working hours.

† Patients with in-hospital mortality rate excluded from analysis.

### Outcomes associated with surgeon resection volumes

The patient characteristics and the co-morbidity burden were similar in patients operated on by the three groups of surgeons with different median yearly resection volumes (*Table 3*). Similarly, the distribution of indications for emergent surgery, the types of colon resections performed, and the median operating time were similar in these groups of patients. There was no difference in tumour stage or the median number of harvested lymph nodes, but the proportion of R_1_ resections differed significantly, with the numerically highest value for surgeons with the highest annual resection volume. However, an adjusted binary logistic regression analysis showed no significant association between resection volumes and R_1_ resections. There was a significant difference in the proportion of complications, with the numerically highest complication rate in patients operated on by surgeons with the highest resection volumes (*Table 4*). Adjusted binary regression analysis confirmed significantly lower odds for complications in patients operated on by surgeons with medium resection volumes, but no such difference was observed for patients operated on by surgeons with the lowest annual resection volumes. The median length of hospital stay, the proportion of reoperations, the need for ICU postoperative care, and the proportion of postoperative mortality rate were similar in patients having their colon resections performed by surgeons with different yearly resection volumes. The proportion of unplanned hospital readmissions, however, differed significantly between the three groups of patients, with almost 19 per cent readmissions in the group of patients operated on by surgeons with the lowest yearly resection volumes. Compared with surgeons with the highest yearly resection volumes, the adjusted OR for unplanned readmissions was six times higher for patients operated on by surgeons with the lowest resection volumes; however, no such difference was observed for the group of patients operated on by surgeons with intermediate resection volumes.

**Table 3:** Patient characteristics and description of surgical procedures in patients who underwent emergent colon cancer resections performed by surgeons with varying median yearly resection volumes

	Surgeons performing			*P*
	<4.11 resections/year (*n* = 80)	4.11–8.40 resections/year (*n* = 96)	>8.40 resections/year (*n* = 59)	
**Sex**				0.190
Male	45 (56.3)	43 (44.8)	34 (57.6)	
Female	35 (43.8)	53 (55.2)	25 (42.4)	
Age (years), median (i.q.r.)	78 (66–83)	78 (66–83)	76 (70–83)	0.501
BMI (kg/m^2^), median (i.q.r.)	23.9 (21.5–27.4)	23.8 (22.0–26.7)	24.8 (21.9–27.7)	0.758
**ASA classification**				
I	10 (12.5)	13 (13.5)	5 (8.5)	0.609
II	30 (37.5)	41(42.7)	30 (50.8)	
III	38 (47.5)	37 (38.5)	21 (35.6)	
IV	2 (2.5)	5 (5.2)	3 (5.1)	
**Charlson co-morbidity index**				
2	31 (38.8)	48 (50.0)	29 (49.2)	0.103
3	18 (22.5)	21 (21.9)	10 (16.9)	
4	9 (11.3)	7 (7.3)	12 (20.3)	
5	3 (3.8)	1 (1.0)	0 (0.0)	
6	14 (17.5)	12 (12.5)	3 (5.1)	
7	5 (6.3)	4 (4.2)	2 (3.4)	
≥8	0 (0)	3 (3.1)	1 (1.7)	
**Indications for emergent surgery**				
Bowel obstruction	63 (78.8)	82 (85.4)	49 (83.1)	0.481
Perforation	12 (15.0)	11 (11.5)	5 (8.5)	
Anaemia/bleeding	5 (6.3)	3 (3.1)	5 (8.5)	
**Surgical procedures**				
Right hemicolectomy	42 (52.5)	44 (45.8)	25 (42.4)	0.643
Colectomy	8 (10.0)	10 (10.4)	11 (18.6)	
Hartmann’s resection	9 (11.3)	6 (6.3)	4 (6.8)	
Sigmoid resection	8 (10.0)	21 (21.9)	9 (15.3)	
Left hemicolectomy	10 (12.5)	12 (12.5)	8 (13.6)	
Transverse colon resection	2 (2.5)	2 (2.1)	2 (3.4)	
Low anterior resection	1 (1.3)	1 (1.0)	0 (0)	
Resections outside regular working hours	36 (45.0)	32 (33.3)	24 (40.7)	0.277
Operating time (min), median (i.q.r.)	196 (143–229)	182 (150–239)	182 (155–245)	0.907
Bleeding (ml), median (i.q.r.)	175 (50–300)	150 (100–250)	200 (100–300)	0.968
Stoma formation*				
Protective stoma	2/39 (5.1)	3/52 (5.8)	3/37 (8.1)	0.851
Permanent stoma	23/39 (59.0)	26/52 (50.0)	12/37 (32.4)	0.062
Any stoma	25/39 (64.1)	29 (55.8)	15 (40.5)	0.113
Lymph node yield, median (i.q.r.)	28 (21–36)	26 (18–38)	28 (20–37)	0.609
Lymph node yield >12	79 (98.8)	91 (94.8)	56 (94.9)	0.334
**R_1_ resections**	2 (2.5)	1 (1.0)	5 (8.5)	0.040
Regression analysis†, OR (95% c.i.)	0.2 (0.0,1.5)	0.1 (0.0,1.1)	Reference	–
**Tumour stage**				
I	1 (1.3)	0 (0)	1 (1.7)	0.773
II	30 (37.5)	33 (34.4)	25 (42.4)	
III	37 (46.3)	47 (49.0)	27 (45.8)	
IV	12 (15.0)	16 (16.7)	6 (10.2)	

Values are *n* (%) unless otherwise indicated. i.q.r., interquartile range; OR, odds ratio.

* Only patients with left-sided tumours included in analysis.

† Binary regression analysis with adjustments for tumour stage, indication for emergent surgery, and resections performed outside regular working hours.

**Table 4:** Postoperative complications and outcome in patients who underwent colon resections performed by surgeons with varying median yearly resection volumes

	Surgeons performing			*P*
	<4.11 resections/year (*n* = 80)	4.11–8.40 resections/year (*n* = 96)	>8.40 resections/year (*n* = 59)	
**Clavien–Dindo classification of complications**				
No complication	35 (43.8)	58 (60.4)	19 (32.2)	0.029
II	24 (30.0)	25 (26.0)	24 (40.7)	
III	11 (13.8)	3 (3.1)	8 (13.6)	
IV	4 (5.0)	5 (5.2)	5 (8.5)	
V	6 (7.5)	5 (5.2)	3 (5.1)	
**Any complication**	45 (56.3)	38 (39.6)	40 (67.8)	0.002
Regression analysis*, OR (c.i.)	0.6 (0.3,1.2)	0.3 (0.1,0.6)	Reference	–
Reoperations	11 (13.8)	5 (5.2)	8 (13.6)	0.109
ICU care	11 (13.8)	11 (11.5)	10 (16.9)	0.625
Length of hospital stay (days), median (i.q.r.)	12 (8–22)	12 (8–18)	14 (10–24)	0.089
**Readmissions†**	14/74 (18.9)	6/91 (6.6)	2/56 (3.6)	0.006
Regression analysis*, OR (c.i.)	6.4 (1.3,30.5)	1.8 (0.3,9.8)	Reference	–
30-day mortality rate	6 (7.5)	4 (4.2)	3 (5.1)	0.619
90-day mortality rate	6 (7.5)	7 (7.3)	5 (8.5)	0.962

Values are *n* (%) unless otherwise indicated. OR, odds ratio; ICU, intensive care unit; i.q.r., interquartile range.

* Binary regression analysis with adjustments for ASA classification, tumour stage, indication for emergent surgery, and surgery performed outside regular working hours.

† Patients with in-hospital mortality rate excluded from analysis.

## Discussion

This study assessed the influence of surgical specialization and surgeon resection volume in patients who underwent emergent resections for colon cancer. The main observation was that emergent resections for colon cancer were performed with similar quality by CS and NCS, especially ACS, with equivalent oncological quality and similar rates of complications and postoperative mortality rate. GS appear to have less favourable results, with more frequent postoperative complications, reoperations, and readmissions.

The proportion of postoperative complications after emergent colon cancer resections in the present study was 52 per cent, which is similar to the complication rates reported in previous studies^5,8,10,13,20^. Although the SCRCR is a validated registry, the observation regarding misclassification of complications in the cohort suggests that studies based on national or regional registries may be burdened with errors in the detection and registration of complications. Consequently, comparisons of complications between studies should be made with caution. The complication rates of patients operated on by NCS, and the subgroup of ACS, were equivalent to that of patients operated on by CS. However, surgeons who do not primarily work in colorectal or acute care surgery had significantly more complications, which was suggested by an adjusted OR that was 2.5 times higher than that of ACS. It is possible that the less advantageous outcome for patients operated on by GS is a result of less frequent exposure to both colorectal and emergent surgery.

The proportion of complications differed significantly in patients operated on by surgeons with various annual resection volumes, and surprisingly the numerically highest complication rate was observed for surgeons with the highest resection volumes. This observation was not explained by differences in clinical features of patients or the surgical procedures, as the adjusted OR for complications differed significantly in comparison with surgeons with intermediate resection volumes.

The observed mortality rates in the present study were similar to those reported for specialized CS at tertiary centres^8,13^, high-volume centres^14,21^, and centres that fulfil national caseload requirements^22^, suggesting that emergent resections for colon cancer can be safely performed at secondary care hospitals. This is important, as most studies on the effect of surgical specialization and resection volume are conducted at large tertiary centres, but, in Sweden, and possibly also in many other countries, the vast majority (80 per cent) of colon cancer resections are performed at secondary care hospitals^15^. The proportion of postoperative mortality rate in the present study was equivalent in patients operated on by CS and NCS, and the subgroup of patients operated on by ACS. The mortality rate of patients operated on by GS was numerically higher, but did not differ significantly compared with patients operated on by CS and ACS. Furthermore, the mortality rates were similar in patients operated on by surgeons with different annual resection volumes.

For policymakers and healthcare institutions, elimination of variations in healthcare quality represents a matter of significant interest. As several studies report a superior outcome among patients undergoing elective surgery in high-volume surgical units by surgeons who perform a large number of procedures^7,23–25^, this has led to an increasing centralization of colorectal surgery to high-volume centres^26^. Although morbidity and mortality rates after elective colon resections are lower in patients operated on at high-volume centres or by specialized surgeons with a high caseload, extrapolating the outcome from elective surgical cases to emergent surgical cases is inadequate. This is due to the fact that the surgical management of patients who are critically ill, and in a poor physiological condition, is a different and a more complex challenge. Additional surgical skills need to complement those required for elective oncological surgery to achieve good postoperative results after emergent resections. Although there are reports proposing that high-volume specialized CS have lower morbidity and mortality rates after emergent resections for colon cancer^8^, there are several studies that support the observations in our study, suggesting similar outcomes for patients having emergent colon resection performed by CS and NCS^9–14^.

The indicators of quality for oncological surgery such as lymph node yield and R_0_ resections were similar in patients undergoing emergent colon resections performed by CS and NCS. This suggests that the quality in oncological surgical technique was equally good regardless of surgical specialization, but studies of long-term and cancer-free survival would be needed to confirm this observation.

The proportions of readmissions and re-interventions after colon cancer surgery may be useful indicators of the quality of surgical care. In the present study, the proportion of reoperations was lowest in patients operated on by ACS, which differed significantly from that of patients operated on by GS. The overall rate of 30-day readmissions in the present study was 8.5 per cent, which compares favourably with other reports on unplanned readmissions after emergent colorectal surgery^27,28^. Compared with CS, the adjusted ORs for readmissions were significantly higher in patients operated on by NCS, and the subgroup of patients operated on by GS, but no such difference was observed for patients operated on by ACS. Furthermore, there was a significant association between unplanned readmissions and colon resections performed by surgeons with the lowest annual resection volume. The reason for the large differences in readmissions is unknown, but possibly represents differences in patient characteristics^29^ or the general quality of care rather than in the quality of the surgical procedure itself, although reports have concluded that the readmission rate is a poor proxy for quality of care^30^. Previous studies have suggested that 30-day readmissions are strongly associated with postoperative complications^31^, but this hypothesis was not confirmed by the observations in the present study, as patients operated on by ACS had the numerically lowest rate of complications, but a rate of unplanned readmissions that was significantly higher than that of patients operated on by CS. Furthermore, it has been suggested that the main reason for unplanned readmissions is occurrences that happen after discharge^32^, which makes them difficult to predict and prevent.

The effect of surgical specialization should ideally be studied in well designed randomized controlled studies, but such studies would be difficult to perform. Bias due to timing of the surgical procedure caused by differences in the severity of the condition of the patients may significantly affect the outcome of emergent colon cancer resections. The most critically ill patients will require immediate surgery, even during the night, when a CS may not be available at many surgical units. Furthermore, less critically ill patients can often wait until the following morning and have emergent resections performed by a CS. Thus, it is possible that CS manage less critically ill patients and may therefore in some studies have more favourable postoperative outcomes than surgeons with other specialties. This perception is supported by a study from a tertiary centre in Norway. They reported that only 7 per cent of emergent colorectal cancer resections were performed by specialized gastrointestinal surgeons at night, which was significantly lower than the proportion of emergent resections performed by this group of surgeons during the day^33^.

It is often problematic to interpret the effect of surgical specialization and resection volumes on postoperative outcome, as studies are heterogeneous and include various proportions of patients with rectal cancer and/or benign disease. The homogeneity of the study population in the present study with no patients with rectal cancer or benign disease is therefore a strength. Another strength is that the study was conducted at a secondary care hospital. As the majority of colon cancer resections in Sweden are performed at secondary care hospitals, it is important to be able to compare results with those from tertiary high-volume centres. The limitations of the study include its retrospective nature, with such studies generally being inferior to prospective trials regarding precision and validity. Another potential limitation is the single-centre cohort study design, as local variations within the groups of surgeons and differences in the patient selection or indications for emergent surgery may influence the generalizability, and comparisons with other studies must therefore be made with caution. Additionally, a potential limitation of all similar studies is that the definitions of surgical specializations may vary. Consequently, conclusions may not be universal, and comparisons have to be made cautiously. In Sweden, colorectal surgery is not a formally acknowledged specialty, but all CS in the present study work at the departments’ colorectal unit and manage patients with colon cancer daily. Many of the surgeons in the present study do rotations at the colorectal and the acute care units, which may contribute to the comparably good results. Another limitation is that the group of patients who underwent emergent colon resections performed by general surgeons, was a relatively small and heterogenic group, and conclusions regarding these results should be made with caution.

Based on the observations in this study it can be summarized that emergent resections for colon cancer can be performed at secondary care hospitals, with complication and mortality rates comparable to those of tertiary high-volume units. Furthermore, in comparison with specialized CS, emergent colon resections were performed with equivalent quality by NCS, especially ACS, with similar rates of morbidity and mortality and similar oncological quality. Patients having colon resections for cancer performed by GS had a less favourable postoperative outcome, with more frequent complications, reoperations, and unplanned readmissions, possibly due to less frequent exposure of GS to both colorectal and emergent surgery. To improve postoperative morbidity and mortality rates for patients undergoing emergent colon resections for cancer, regular rotations at colorectal and acute care units for surgeons performing emergent surgery may be needed to achieve and maintain competence in colorectal and acute care surgery.

## Supplementary Material

zrad033_Supplementary_Data

## Data Availability

Due to their proprietary nature and ethical concerns, supporting data cannot be made openly available. Further information about the data and conditions for access are available from the authors on request.
