# Supplementary material for: Effects of surgical specialization and surgeon resection volume on postoperative complications and mortality rate after emergent colon cancer resection
Source: BJS Open. 2023 May 9;7(3):zrad033. doi: 10.1093/bjsopen/zrad033 (PMC10167707; doi:10.1093/bjsopen/zrad033)
Supplement: zrad033_Supplementary_Data [file zrad033_supplementary_data.docx]

**Effects of surgical specialization and surgeon resection volume on postoperative complications and mortality after emergent colon cancer resection**

**Authors**: Jenny Engdahl^1^, Astrid Öberg^1^, Henrik Bergenfeldt^2^, Marcus Edelhamre^1^, Tomas Vedin^2^, Sandra Bech-Larsen^1^, Stefan Öberg^1^.

**^1^Affiliation:** The Department of Surgery, Helsingborg Hospital,

Clinical Sciences Lund, Lund University, Sweden.

**^2^Affiliation:** The Department of Surgery, Skåne University Hospital,

Clinical Sciences Lund, Lund University, Sweden.

**Corresponding author.** Name and address **ORCID ID**; **Twitter**

**J**enny Engdahl

The Department of Surgery, Helsingborg Hospital,

Charlotte Yhlens gata 10, 252 23 Helsingborg

E-mail: [jenny.engdahl-severin@skane.se](mailto:jenny.engdahl-severin@skane.se)

ORCID ID: 0000-0003-0737-6433

**Supplementary Materials - Index**

| **Supplementary Tables** |  |
| --- | --- |
| Table S1 | *page 2* |
| Table S2 | *page 4* |

**Table S1. Patient characteristics and descriptions of the surgical procedures in patients who underwent emergent colon cancer resections performed by colorectal surgeons and non-colorectal surgeons.**

|  | Resections performed by | | p-value |
| --- | --- | --- | --- |
|  | Colorectal surgeons  n=99 | Non-colorectal surgeons n=136 |  |
| Age (years) | 76 (68-83) | 77 (68-84) | 0.659 |
| Gender (F/M) | 47:52 | 66:70 | 0.873 |
| BMI (kg/m^2^) | 24.3 (21.9-27.5) | 23.8 (21.7-27.3) | 0.539 |
| ASA-classification | | | |
| - I | 10 (10.1%) | 18 (13.2%) | 0.862 |
| - II | 43 (43.4%) | 58 (42.6%) |  |
| - III | 41 (41.4%) | 55 (40.4%) |  |
| - IV | 5 (5.1%) | 5 (3.7%) |  |
| Charlson Comorbidity Index | | | |
| - 2 | 48 (48.5%) | 60 (44.1%) | 0.511 |
| - 3 | 19 (19.2%) | 30 (22.1%) |  |
| - 4 | 14 (14.1%) | 14 (10.3%) |  |
| - 5 | 1 (1.0%) | 3 (2.2%) |  |
| - 6 | 8 (8.1%) | 21 (15.4%) |  |
| - 7 | 5 (5.1%) | 6 (4.4%) |  |
| - =>8 | 4 (4.0%) | 2 (1.5%) |  |
| Indications for surgery | | | |
| - Obstruction | 81 (81.8%) | 113 (83.1%) | 0.949 |
| - Perforation | 12 (12.1%) | 16 (11.8%) |  |
| - Anemia/bleeding | 6 (6.1%) | 7 (5.1%) |  |
| Surgical procedures | | | |
| - Right hemicolectomy | 50 (50.5%) | 61 (44.9%) | 0.884 |
| - Sigmoid resection | 15 (15.2%) | 23 (16.9%) |  |
| - Colectomy | 14 (14.1%) | 15 (11.0%) |  |
| - Left hemicolectomy | 10 (10.1%) | 20 (14.7%) |  |
| - Hartmanns resection | 7 (7.1%) | 12 (8.8%) |  |
| - Transverse colon resection | 2 (2.0%) | 4 (2.9%) |  |
| - Low anterior resection | 1 (1.0%) | 1 (0.7%) |  |
| Resections outside regular working hours | 39 (39.4%) | 53 (39.0%) | 0.948 |
| Operating time | 180 (150 - 241) | 191 (152 - 230) | 0.843 |
| Peroperative bleeding (ml) | 200 (100-300) | 150 (100-250) | 0.383 |
| Lymph node yield | 27 (21-38) | 27 (20-36) | 0.411 |
| Lymph node yield > 12 | 95 (96.0%) | 131 (96.3%) | 0.886 |
| R_1_-resection  - regression analysis^a^ | 6 (6.1%) | 2 (1.5%) | 0.061 |
|  | 3.9 (0.7 – 20.9) | Reference | - |
| Stoma formation^b^ | | | |
| - Permanent stoma | 21/52 (40.4%) | 40/76 (52.6%) | 0.173 |
| - Protective stoma | 6/52 (11.5%) | 2/76 (2.6%) | 0.041 |
| - Any stoma | 27/52 (51.9%) | 42/76 (55.3%) | 0.710 |
| Tumor stage | | | |
| - I | 2 (2.0%) | 0 (0%) | 0.405 |
| - II | 38 (38.4%) | 50 (36.8%) |  |
| - III | 45 (45.5%) | 66 (48.5%) |  |
| - IV | 14 (14.1%) | 20 (14.7%) |  |

Continuous variables are presented as medians and 25^th^ – 75^th^ percentiles and

categorical data are presented as numbers (%).

ASA = American Society of Anesthesiologist. BMI = Body Mass Index.

^a^ Binary regression analysis with adjustments for tumor stage, indication for emergent surgery and resections performed outside regular working hours. (Odds ratio (95% confidence interval)).

^b^ Only patients with left-sided tumors included in analysis.

**Table S2. Postoperative complications and outcome after emergent colon cancer resections performed by colorectal surgeons and non-colorectal surgeons.**

|  | Resections performed by | | p-value |
| --- | --- | --- | --- |
|  | Colorectal surgeons  n=99 | Non-colorectal surgeons n=136 |  |
| Clavien-Dindo classification of complications | | | |
| - No complication | 45 (45.5%) | 67 (49.3%) | 0.741 |
| - II | 33 (33.3%) | 40 (29.4%) |  |
| - III | 10 (10.1%) | 12 (8.8%) |  |
| - IV | 7 (7.1%) | 7 (5.1%) |  |
| - V | 4 (4.0%) | 10 (7.4%) |  |
| Any complication | 54 (54.5%) | 69 (50.7%) | 0.564 |
| Care at ICU  - regression analysis^a^ | 18 (18.2%) | 14 (10.3%) | 0.082 |
|  | Reference | 0.5 (0.2-1.1) | - |
| Reoperations | 12 (12.1%) | 12 (8.8%) | 0.410 |
| Length of hospital stay (days) | 13 (9-22) | 12 (8 – 18) | 0.257 |
| Readmissions^b^  - regression analysis^a^ | 4/95 (4.2%) | 18/126 (14.3%) | 0.013 |
|  | Reference | 4.2 (1.3-13.2) | 0.016 |
| 30-day mortality | 3 (3.0%) | 10 (7.4%) | 0.152 |
| 90-day mortality | 8 (8.1%) | 10 (7.4%) | 0.836 |

Categorical data are presented as numbers (%).

ICU = Intensive Care Unit.

^a^ Binary regression analysis with adjustments for ASA-classification, tumor stage, indication for emergent surgery and surgery performed outside regular working hours. (Odds ratio (95% confidence interval)).

^b^ Patients with in-hospital mortality excluded from analysis.
